# Using carrot centromeric repeats to study karyotype relationships in the genus *Daucus* (Apiaceae)

**DOI:** 10.1186/s12864-021-07853-2

**Published:** 2021-07-06

**Authors:** Dariusz Kadluczka, Ewa Grzebelus

**Affiliations:** Department of Plant Biology and Biotechnology, Faculty of Biotechnology and Horticulture, University of Agriculture in Krakow, al. 29 Listopada 54, 31-425 Krakow, Poland

**Keywords:** Crop wild relatives, Cytotaxonomy, Karyomorphology, Molecular cytogenetics, Plant chromosomes

## Abstract

**Background:**

In the course of evolution, chromosomes undergo evolutionary changes; thus, karyotypes may differ considerably among groups of organisms, even within closely related taxa. The genus *Daucus* seems to be a promising model for exploring the dynamics of karyotype evolution. It comprises some 40 wild species and the cultivated carrot, a crop of great economic significance. However, *Daucus* species are very diverse morphologically and genetically, and despite extensive research, the taxonomic and phylogenetic relationships between them have still not been fully resolved. Although several molecular cytogenetic studies have been conducted to investigate the chromosomal structure and karyotype evolution of carrot and other *Daucus* species, detailed karyomorphological research has been limited to carrot and only a few wild species. Therefore, to better understand the karyotype relationships within *Daucus*, we (1) explored the chromosomal distribution of carrot centromeric repeats (CentDc) in 34 accessions of *Daucus* and related species by means of fluorescence in situ hybridization (FISH) and (2) performed detailed karyomorphological analysis in 16 of them.

**Results:**

We determined the genomic organization of CentDc in 26 accessions of *Daucus* (belonging to both *Daucus* I and II subclades) and one accession of closely related species. The CentDc repeats were present in the centromeric regions of all chromosomes of 20 accessions (representing 11 taxa). In the other *Daucus* taxa, the number of chromosome pairs with CentDc signals varied depending on the species, yet their centromeric localization was conserved. In addition, precise chromosome measurements performed in 16 accessions showed the inter- and intraspecific karyological relationships among them.

**Conclusions:**

The presence of the CentDc repeats in the genomes of taxa belonging to both *Daucus* subclades and one outgroup species indicated the ancestral status of the repeat. The results of our study provide useful information for further evolutionary, cytotaxonomic, and phylogenetic research on the genus *Daucus* and may contribute to a better understanding of the dynamic evolution of centromeric satellites in plants.

**Supplementary Information:**

The online version contains supplementary material available at 10.1186/s12864-021-07853-2.

## Background

Chromosomes undergo evolutionary changes in the course of evolution; thus, karyotypes may differ considerably among groups of organisms, even within closely related taxa. Hence, the study of chromosomes by means of karyotypic features, including the number, size, centromere position, number and position of secondary constrictions, symmetry, and banding patterns of the chromosome complement, has been widely used in taxonomy (cytotaxonomy), systematics, and phylogeny, thus greatly contributing to our understanding of evolutionary processes. It has also been confirmed that repetitive DNA sequences are tightly associated with chromosome evolution in plants [1–3].

A substantial portion of plant genomes are composed of various types of repetitive DNA sequences, classified as tandem or dispersed, according to the genomic organization of their repeat units. The dispersed repeats are scattered throughout the genome (transposable elements), whereas tandem repeats appear in the form of large arrays consisting of thousands or millions of monomers, comprising microsatellites, minisatellites, and satellite DNA [4, 5]. Unlike low-copy-number sequences, repetitive elements are highly variable and evolve more rapidly, leading to changes in the abundance and chromosomal distribution of their copies. Furthermore, due to their high copy number and tendency to cluster, they are excellent probes for fluorescence in situ hybridization (FISH), a powerful molecular cytogenetic technique, providing valuable information on their physical localization, thus making them advantageous for comparative studies concerning evolutionary relationships between species [6, 7]. Of these, satellite DNA has greatly contributed to our knowledge on chromosome and genome evolution, as well as the phylogeny of species. This class of repeats is preferentially associated with specific chromosomal segments, most frequently found at centromeric, pericentromeric, and subtelomeric regions but also at intercalary positions [4, 5, 8]. Chromosomal sites rich in satellite DNA usually exhibit a unique banding pattern, which makes them ideal as cytogenetic markers for the identification of individual chromosomes; therefore they are useful for karyotype descriptions [9–14]. FISH with satellite DNA-based probes has also been successfully applied for the understanding of chromosomal evolution in several agronomically important plant species, including sugar beet [8], maize [15], radish [16], common bean [17], spinach [18], and quinoa [19].

Carrot (*Daucus carota *subsp.* sativus* Hoffm.), belonging to the large and complex family Apiaceae, is the most significant member of the genus *Daucus*, being a major source of vitamin A precursors (*α*- and *β*-carotene) in the human diet [20]. The genus comprises some 40 wild species known to be morphologically and genetically diverse. For this reason, despite numerous research efforts at various levels (morphological, anatomical, and molecular), the taxonomic delimitation and phylogenetic relationships between them have still not been fully resolved [21–28]. The correlation of the *Daucus* taxonomy with its phylogeny is a challenging task because the clades inferred from molecular data have no obvious morphological synapomorphies allowing the recognition of their taxa. On the basis of recent molecular studies using plastid and nuclear DNA sequences *Daucus* species were divided into two subclades: *Daucus* I and *Daucus* II, of which *Daucus* I groups the wild ancestor of the cultivated carrot and its subspecies, several Mediterranean *Daucus* species, and some members of other genera (*Athamanta*, *Pachyctenium*, *Pseudorlaya*, *Tornabenea*), whereas *Daucus* II includes the remaining *Daucus* members, along with its American and Australian representatives [23, 25]. A recent reevaluation of *Daucus* by Banasiak et al. [26], in which the nuclear ribosomal DNA ITS and three chloroplast markers were used, has expanded the genus to include the following taxa: *Agrocharis* Hochst. (four species), *Melanoselinum* Hoffm. (one species), *Monizia* Lowe (one species), *Pachyctenium* Maire & Pamp. (one species), *Pseudorlaya* (Murb.) Murb. (two species), *Rouya* Coincy (one species), *Tornabenea* Parl. (six species), *Athamanta dellacellae* E.A. Durand & Barratte, and *Cryptotaenia elegans* Webb ex Bolle.

The great diversity of *Daucus* species makes it a promising model for cytotaxonomic and evolutionary research. To date, several molecular cytogenetic studies have been conducted to investigate the chromosomal structure and karyotype evolution of carrot and other *Daucus* [29–34]. Nonetheless, detailed karyomorphological studies in *Daucus* have been limited to carrot [29, 32, 35–38] and only a few wild species [29].

When dealing with chromosomes of different *Daucus* species, it is often difficult to obtain metaphase spreads suitable for precise measurements. This is due to the relative morphological uniformity of the chromosomes, in which the position of the primary constrictions is not always possible to unequivocally determine. In carrot, however, this obstacle has recently been overcome through the identification of a carrot centromeric repeat, named CentDc, which is typically composed of four 39–40-bp monomers that vary slightly in sequence [30, 39]. Consequently, a consensus sequence derived from these monomers was used as a FISH probe, along with some other repetitive probes, for hybridization to metaphase chromosomes of carrot, enabling detailed karyotype measurements and differentiation of its individual chromosomes [32]. In addition to *Daucus carota*, comparative in silico analysis was conducted on five other *Daucus* species, indicating the presence of CentDc-like sequences in three of them, whereas the two remaining ones were further analyzed by FISH to confirm the absence of these repeats [33]. These findings suggest the hypothesis that carrot centromeric repeats are widespread in the genus *Daucus* and that their chromosomal distribution can be examined by molecular cytogenetics. However, the detailed and comprehensive comparative FISH mapping of carrot centromeric sequences in *Daucus* has not been reported before.

In this study, we aimed to address how carrot centromeric repeats have evolved; therefore, we employed a FISH-based approach to explore the chromosomal distribution of these repeats in 34 accessions of *Daucus* and related taxa. Subsequently, we identified taxa that – like carrot – carry CentDc repeats in the primary constrictions of all chromosomes, which, in turn, enabled us to take precise karyotype measurements. Moreover, these data allowed us to discuss the relationships among *Daucus* species based on their karyotype features.

## Results

### Comparative FISH mapping of CentDc repeats

For comparative FISH analysis, we selected 34 accessions of *Daucus* (belonging to both *Daucus* I and II subclades) and related species (Table 1).

**Table 1:** List of *Daucus* accessions and related species (outgroups) used in this study

Taxon^a^	2*n*	Seed source^b^/Accession no.^c^	Country of origin
*Daucus* I subclade			
*D. aureus*	22	USDA/PI 295854	Israel
*D. aureus*	22	USDA/PI 319403	Israel
*D. carota* subsp. *capillifolius*	18	USDA/PI 279764	Libya
*D. carota* subsp. *capillifolius*	18	USDA/Ames 30198	Tunisia
*D. carota* subsp. *carota*	18	USDA/PI 274297	Pakistan
*D. carota* subsp. *carota*	18	USDA/PI 478369	China
*D. carota* subsp. *carota*	18	USDA/PI 478861	France
*D. carota* subsp. *carota*	18	USDA/PI 652393	Turkey
*D. carota* subsp. *gummifer*	18	USDA/PI 478883	France
*D. carota* subsp. *gummifer*	18	USDA/Ames 26383	Portugal
*D. carota* subsp. *maximus*	18	USDA/Ames 26408	Portugal
*D. carota* subsp. *sativus*	18	RZ/DH1*	The Netherlands
*D. carota* subsp. *sativus*	18	Commercial/‘Dolanka’**	Poland
*D. carota* subsp. *sativus*	18	Commercial/‘Amsterdam’**	Poland
*D. crinitus*	22	USDA/PI 652414	Portugal
*D. muricatus*	22	USDA/PI 295863	Spain
*D. muricatus*	22	USDA/Ames 29090	Tunisia
*D. pumilus*	16	USDA/PI 662301	Tunisia
*D. rouyi*	20	USDA/PI 674284	Tunisia
*D. sahariensis*	18	USDA/Ames 29096	Tunisia
*D. sahariensis*	18	USDA/Ames 29097	Tunisia
*D. syrticus*	18	USDA/Ames 29108	Tunisia
*Daucus* II subclade			
*D. conchitae*	22	USDA/Ames 25835	Turkey
*D. glochidiatus*	44	USDA/PI 285038	Australia
*D. involucratus*	22	USDA/PI 652332	Greece
*D. involucratus*	22	USDA/PI 652355	Turkey
*D. littoralis*	20	USDA/PI 295857	Israel
* D. pusillus*	22	USDA/PI 349267	Uruguay
Outgroups			
*Ammi visnaga*	20	IPK/AMMI 25	Germany
*Astrodaucus littoralis*	20	USDA/PI 277064	Azerbaijan
*Caucalis platycarpos*	20	USDA/PI 649446	Germany
*Orlaya daucoides*	16	USDA/PI 649477	Turkey
*Torilis arvensis*	12	USDA/PI 649391	Syria
*T. arvensis*	12	USDA/PI 649394	Turkey

^a^ Taxonomic classification according to [25, 26]

^b^ *IPK,* Leibniz Institute of Plant Genetics and Crop Plant Research (IPK), Gatersleben, Germany; *RZ,* Rijk Zwaan vegetable breeding company, Lier, the Netherlands; *USDA,* USDA-ARS North Central Regional Plant Introduction Station (NCRPIS), Ames, Iowa, USA

^c^ *Ames*, Ames numbers are assigned to carrots and other Apiaceae maintained at the NCRPIS; *PI*, USDA Plant Introduction numbers are permanent numbers assigned to germplasm accessions in the National Plant Germplasm System (NPGS); (*) *DH1*, a doubled haploid orange Nantes type carrot breeding line; (**) carrot cultivars

**Fig. 1:**
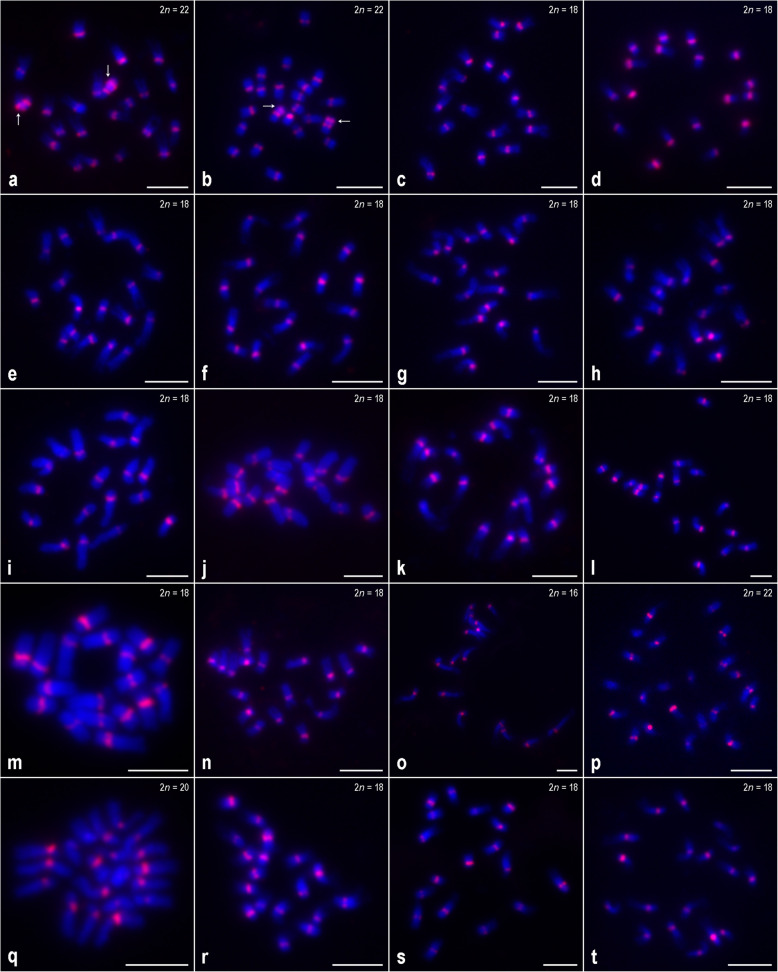
FISH mapping of the CentDc probe (red signals) to metaphase chromosomes of *Daucus* taxa. **a** *D. aureus* [PI 295854]; **b** *D. aureus* [PI 319403]; **c** *D. carota* subsp. *capillifolius* [PI 279764]; **d** subsp. *capillifolius* [Ames 30198]; **e** subsp. *carota* [PI 274297]; **f** subsp. *carota* [PI 478369]; **g** subsp. *carota* [PI 478861]; **h** subsp. *carota* [PI 652393]; **i** subsp. *gummifer* [PI 478883]; **j** subsp. *gummifer* [Ames 26383]; **k** subsp. *maximus*; **l** subsp. *sativus* (‘Amsterdam’); **m** subsp. *sativus* (‘Dolanka’); **n** subsp. *sativus* (DH1); **o** *D. pumilus*; **p** *D. pusillus*; **q** *D. rouyi*; **r** *D. sahariensis* [Ames 29096]; **s** *D. sahariensis* [Ames 29097]; **t** *D. syrticus*. *Arrows* in (**a**) and (**b**) indicate additional CentDc signals in the interstitial regions of the long arms of the chromosomes. Scale bar = 5 μm

**Fig. 2:**
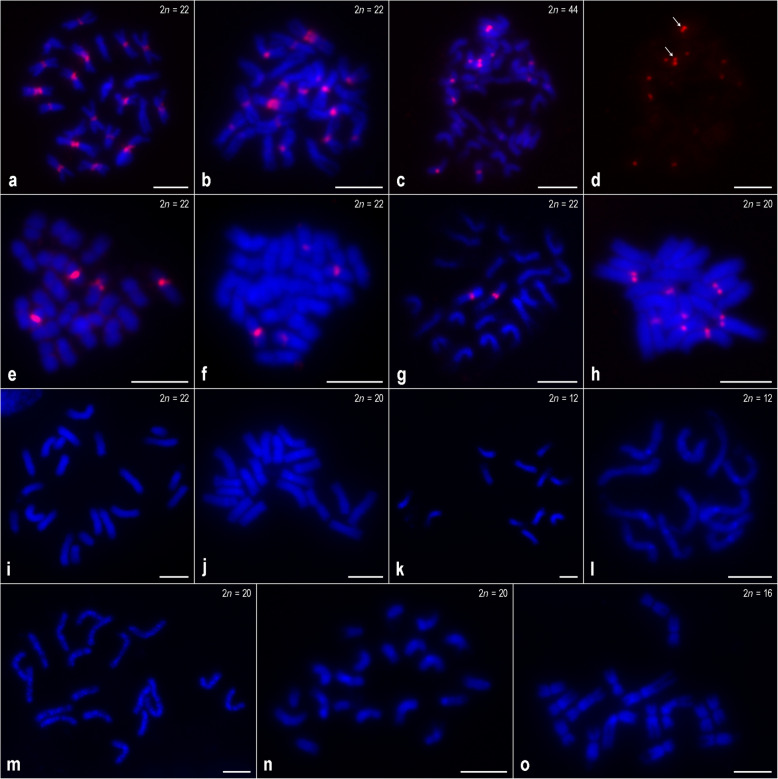
FISH mapping of the CentDc probe (red signals) to metaphase chromosomes of *Daucus* taxa and related species. **a** *D. muricatus* [PI 295863]; **b** *D. muricatus* [Ames 29090]; **c** *D. glochidiatus*; **d** FISH signals from subpanel **c**, *arrows* indicate a chromosome pair with distinctly strong signals; **e** *D. involucratus* [PI 652332]; **f** *D. involucratus* [PI 652355]; **g** *D. conchitae*; **h** *Aastrodaucus littoralis*; **i** *D. crinitus*; **j** *D. littoralis*; **k** *Torilis arvensis* [PI 649391]; **l** *T. arvensis* [PI 649394]; **m** *Ammi visnaga*; **n** *Caucalis platycarpos*; **o** *Orlaya daucoides*. **i**–**o** These species did not produced CentDc signals after FISH. Scale bar = 5 μm

FISH on metaphase chromosome spreads with CentDc (hereinafter referred to as a 36-nucleotide sequence based on the consensus sequence corresponding to a subrepeat of the original CentDc repeat; see ‘Methods’) were used as a probe and displayed a clear hybridization pattern in 27 out of 34 accessions examined in this study (Fig. 1 and 2a–h). Seven other accessions, representing six taxa, did not show any fluorescence signals, suggesting either the absence or low copy number of CentDc repeats in their genomes. Metaphase chromosomes of these FISH-negative accessions are shown in Fig. 2i–o.

In the case of 20 accessions, representing 11 taxa (10 taxa belonging to the *Daucus* I subclade, 1 taxon belonging to the *Daucus* II subclade), the CentDc probe hybridized to the centromeric regions of all chromosomes (Fig. 1). In each accession, the fluorescence intensity of the FISH signals varied between different chromosomes, indicating differences in copy number of CentDc repeats. However, these differences in the fluorescence intensity were not sufficient to enable the identification of all homologous chromosomes. Among these taxa, both accessions of *D. aureus* (2*n* = 22) had one chromosome pair showing additional signals of CentDc – along with the centromeric ones – observed in the interstitial regions of the long arms (Fig. 1a–b, *arrows*).

The other FISH-positive taxa displayed different hybridization patterns that varied in terms of the number of chromosome pairs with CentDc signals. Among them, both accessions of *D. muricatus* (2*n* = 22) (*Daucus* I subclade) had eight CentDc-carrying chromosome pairs in which the signals were located either in the centromeric or pericentromeric regions (Fig. 2a–b). For *D. glochidiatus* (2*n* = 44) (*Daucus* II subclade), the only polyploid species analyzed here, centromeric signals were revealed on five chromosome pairs, of which one pair was marked by distinctly strong signals (Fig. 2c–d, *arrows*), whereas the remaining chromosomes harbored much weaker signals that were often difficult to detect. On the other hand, both accessions of *D. involucratus* (2*n* = 22) and *D. conchitae* (2*n* = 22) (both species from the *Daucus* II subclade) showed the fewest CentDc signals. In the *D. involucratus* accessions, CentDc repeats hybridized to two chromosome pairs, occupying centromeric regions (Fig. 2e–f), while *D. conchitae* produced CentDc signals on one chromosome pair (Fig. 2g). In the latter, the signals were difficult to score as centromeric; thus, we performed FISH to meiotic chromosomes of that species (see below).

Interestingly, CentDc signals were also found on four chromosome pairs of the outgroup species, *Astrodaucus littoralis* (Fig. 2h), suggesting the ancestral status of this repeat. We were, however, not able to determine whether the signals were centromeric. No signals were observed in the other outgroup species.

To confirm the centromeric position of the CentDc repeats or to visualize them at a greater resolution, FISH was performed on both meiotic chromosomes and chromosomes in mitotic anaphase of selected accessions (Additional file 1: Fig. S1). In some cases, depending on the degree of chromatin condensation, CentDc signals coincided with cytologically recognizable heterochromatic knobs on pachytene chromosomes. The results also showed that at meiotic metaphase I and mitotic anaphase, the signals were located at the most poleward positions, confirming the centromeric specificity of these repeats.

### Karyotype analysis

Accessions selected for detailed karyotype analysis were those that (1) produced CentDc signals in the centromeric regions of all chromosomes and (2) had metaphase spreads containing only well-condensed chromosomes with clearly defined boundaries. The only exception was *Orlaya daucoides*, which, despite being FISH-negative, had chromosomes with a distinct primary constriction (Fig. 2o); therefore, it was also subjected to karyotyping. Although five other accessions met the first criterion (*D. aureus* [PI 295854], *D. carota* subsp. *maximus* [Ames 26408], *D. pumilus* [PI 662301], *D. pusillus* [PI 349267], *D. sahariensis* [Ames 29096]), we failed to obtain a sufficient number of good quality metaphase spreads (the second criterion); hence, they were excluded from karyotyping.

**Fig. 3:**
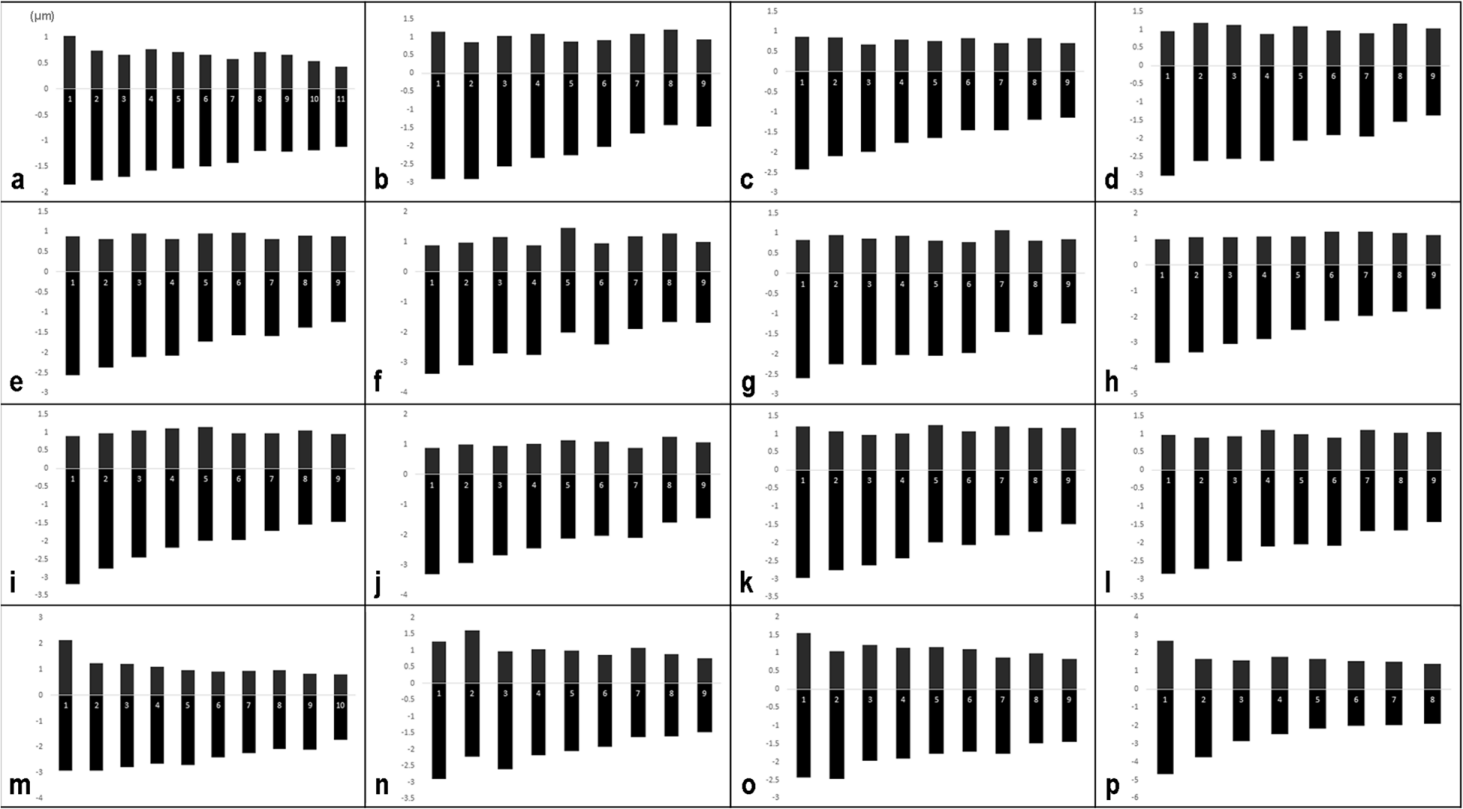
The mean haploid idiograms of the 16 accessions of *Daucus* and *Orlaya daucoides*, which are listed in Table 2. **a** *D. aureus*; **b** *D. carota* subsp. *capillifolius* [PI 279764]; **c** subsp. *capillifolius* [Ames 30198]; **d** subsp. *carota* [PI 274297]; **e** subsp. *carota* [PI 478369]; **f** subsp. *carota* [PI 478861]; **g** subsp. *carota* [PI 652393]; **h** subsp. *gummifer* [PI 478883]; **i** subsp. *gummifer* [Ames 26383]; **j** subsp. *sativus* (‘Amsterdam’); **k** subsp. *sativus* (‘Dolanka’); **l** subsp. *sativus* (DH1); **m** *D. rouyi*; **n** *D. sahariensis*; **o** *D. syrticus*; **p** *O. daucoides*

The detailed karyotype features of the 16 karyotyped accessions (representing 9 taxa) are summarized in Table 2. The mean haploid idiograms of each accession are shown in Fig. 3.

**Table 2:** Karyotype features of the studied accessions

Taxon	Accession	2*n*	KF^a^	THCL^b^ (μm)	CLR^c^ (μm)	CV_CL_^d^	CV_CI_^e^	M_CA_^f^	St^g^
*Daucus aureus*	PI 319403	22	1m + 10sm	23.50	1.54–2.89	17.85	10.23	36.89	3A
*D. carota* subsp. *capillifolius*	PI 279764	18	8sm + 1st	28.67	2.32–4.08	17.68	22.12	34.63	3A
*D. carota* subsp. *capillifolius*	Ames 30198	18	8sm + 1st	22.19	1.89–3.36	18.33	16.94	34.79	3A
*D. carota* subsp. *carota*	PI 274297	18	7sm + 2st	28.95	2.38–4.03	17.26	20.19	34.01	3A
*D. carota* subsp. *carota*	PI 478369	18	1m + 6sm + 2st	24.56	2.07–3.61	16.21	18.24	33.36	2A
*D. carota* subsp. *carota*	PI 478861	18	1m + 4sm + 4st	31.15	2.62–4.33	15.13	26.50	35.89	3A
*D. carota* subsp. *carota*	PI 652393	18	6sm + 3st	25.23	2.04–3.45	15.52	19.38	35.79	3A
*D. carota* subsp. *gummifer*	PI 478883	18	5sm + 4st	33.47	2.72–4.77	17.42	24.31	35.94	3A
*D. carota* subsp. *gummifer*	Ames 26383	18	1m + 5sm + 3st	28.32	2.39–4.20	17.41	18.45	34.06	2A
*D. carota* subsp. *sativus*	‘Amsterdam’	18	7sm + 2st	29.86	2.55–4.16	16.04	24.37	35.73	3A
*D. carota* subsp. *sativus*	‘Dolanka’	18	1m + 8sm	29.90	2.65–4.26	14.70	18.93	31.04	3A
*D. carota* subsp. *sativus*	DH1	18	8sm + 1st	28.08	2.42–3.91	14.38	19.65	34.45	3A
*D. rouyi*	PI 674284	20	2m + 8sm	35.50	2.45–5.12	20.41	14.47	38.74	3A
*D. sahariensis*	Ames 29097	18	2m + 7sm	27.97	2.24–4.20	20.46	13.68	32.28	3A
*D. syrticus*	Ames 29108	18	6m + 3sm	26.86	2.25–4.07	17.66	9.39	26.08	2A
*Orlaya daucoides*	PI 649477	16	2m + 6sm	35.61	3.27–7.73	30.47	12.16	20.55	2A

*Note*: The formulae of the above parameters (CV_CL_, CV_CI_, M_CA_) and the karyotype symmetry classes of Stebbins are given in Additional file 2: Table S1 and Additional file 3: Table S2, respectively

^a^ *KF,* haploid karyotype formula (*m*, metacentric; *sm*, submetacentric; *st*, subtelocentric)

^b^ *THCL,* mean total haploid chromosome length

^c^ *CLR,* Chromosome length range

^d^ *CV*_*CL*_ , Coefficient of variation of chromosome length

^e^ *CV*_*CI*_ , Coefficient of variation of the centromeric index

^f^ *M*_*CA*_ , Mean centromeric asymmetry

^g^ *St,* karyotype symmetry class according to Stebbins [40]

Among the studied accessions, *O. daucoides* had the highest total haploid chromosome length (THCL); however, considering only the genus *Daucus*, the highest THCL was found in *D. rouyi*, while *D. carota* subsp. *capillifolius* [Ames 30198] had the lowest value of this parameter. The longest chromosome was also observed in *O. daucoides*, but in the genus *Daucus*, the longest chromosome occurred in *D. carota* subsp. *gummifer* [PI 478883], which was three times longer than the shortest chromosome that was found in *D. aureus*. The accessions differed in their haploid karyotype formula (KF), even within the same taxa, except *D. carota* subsp. *capillifolius*, whose both accessions shared the same KF. The karyotypes were composed of metacentric, submetacentric and subtelocentric chromosomes, with submetacentric being the most common form of chromosomes, representing 72.6% of all chromosomes.

**Fig. 4:**
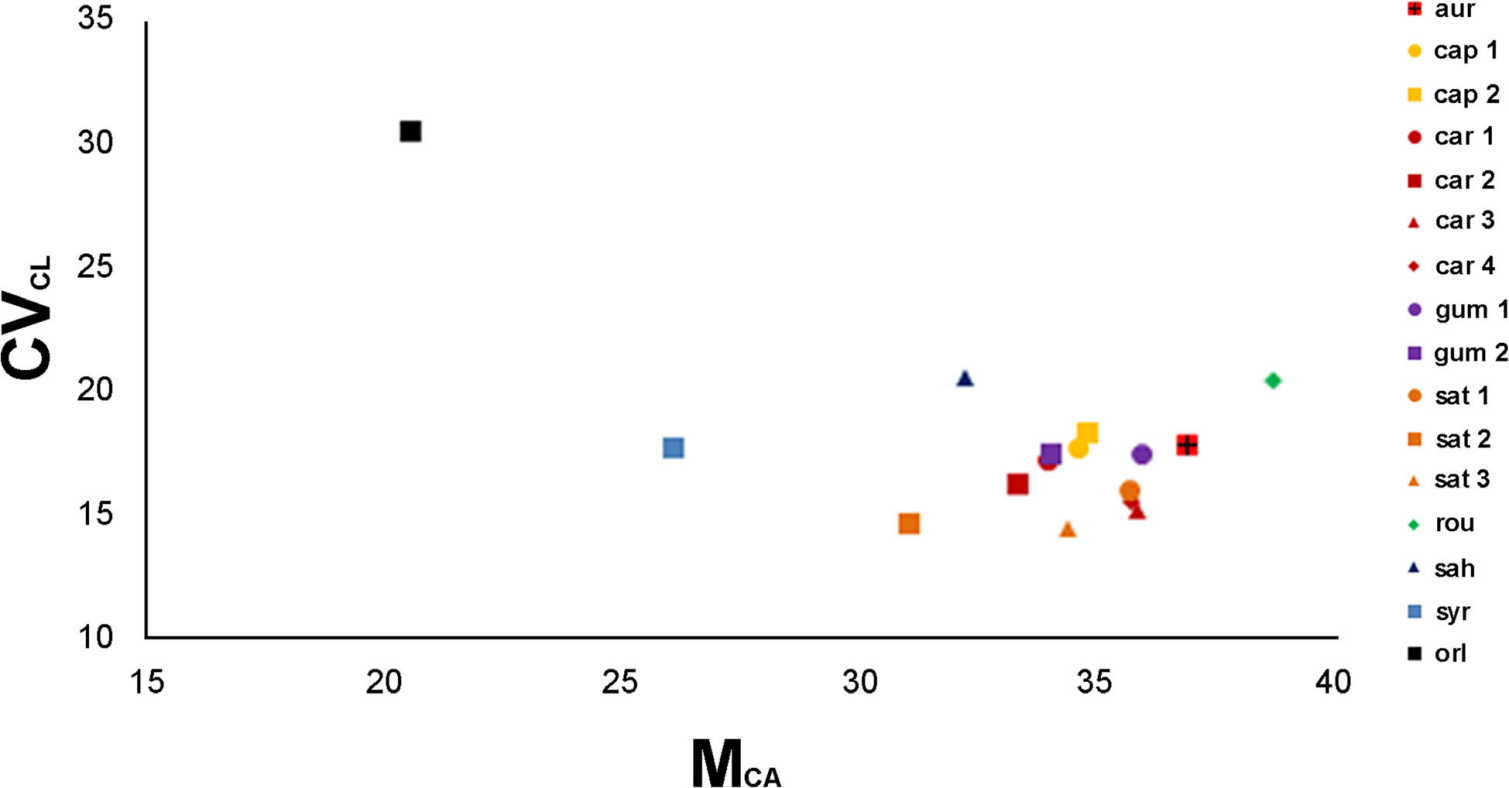
Scatter plot with parameters CV_CL_ vs. M_CA_ illustrating karyotype asymmetry relationships among the 16 accessions of *Daucus* and *Orlaya daucoides*, which are listed in Table 2. *aur* = *D. aureus*; *cap 1* = *D. carota* subsp. *capillifolius* [PI 279764]; *cap 2* = subsp. *capillifolius* [Ames 30198]; *car 1* = subsp. *carota* [PI 274297]; *car 2* = subsp. *carota* [PI 478369]; *car 3* = subsp. *carota* [PI 478861]; *car 4* = subsp. *carota* [PI 652393]; *gum 1* = subsp. *gummifer* [PI 478883]; *gum 2* = subsp. *gummifer* [Ames 26383]; *sat 1* = subsp. *sativus* (‘Amsterdam’); *sat 2* = subsp. *sativus* (‘Dolanka’); *sat 3* = subsp. *sativus* (DH1); *rou* = *D. rouyi*; *sah* = *D. sahariensis*; *syr* = *D. syrticus*; *orl* = *O. daucoides*

Among the genus *Daucus*, *D. sahariensis* and *D. rouyi* exhibited the highest interchromosomal asymmetry, as evidenced by CV_CL_ values, while *D. carota* subsp. *sativus* (DH1) was found to have the most symmetrical karyotype in this regard. Moreover, *D. rouyi* also showed the highest intrachromosomal asymmetry, as indicated by the M_CA_ value, whereas *D. syrticus* had the lowest value of this parameter. CV_CI_ values showed that *D. carota* subsp. *carota* [PI 478861] had the most heterogeneous karyotype in terms of centromere position, while the karyotype of *D. syrticus* was the most homogeneous in this regard. However, when including *O. daucoides* (outgroup), this species was characterized by the most asymmetrical karyotype in terms of interchromosomal asymmetry, but at the same time, it showed the lowest intrachromosomal asymmetry of all karyotyped accessions. Relationships among the examined accessions based on the asymmetry indices are illustrated in Fig. 4. According to the classification of Stebbins [40], the karyotyped accessions were pooled into two classes, namely 2A and 3A, with a predominance of the 3A class (representing 75%).

**Fig. 5:**
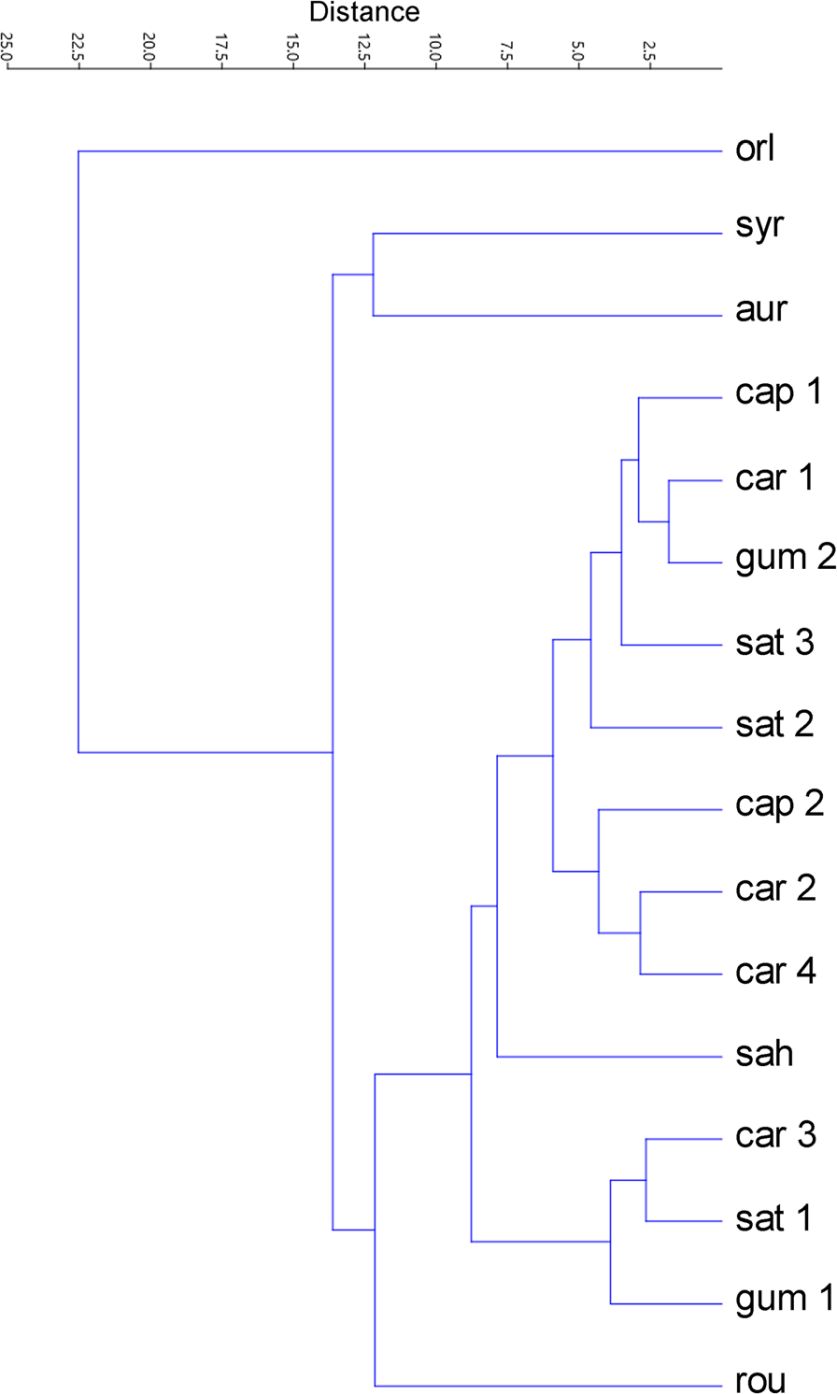
UPGMA dendrogram based on six quantitative parameters (*x*, 2*n*, THCL, M_CA_, CV_CL_, CV_CI_) showing the karyological relationships of the 16 accessions of *Daucus* and *Orlaya daucoides*, which are listed in Table 2. *aur* = *D. aureus*; *cap 1* = *D. carota* subsp. *capillifolius* [PI 279764]; *cap 2* = subsp. *capillifolius* [Ames 30198]; *car 1* = subsp. *carota* [PI 274297]; *car 2* = subsp. *carota* [PI 478369]; *car 3* = subsp. *carota* [PI 478861]; *car 4* = subsp. *carota* [PI 652393]; *gum 1* = subsp. *gummifer* [PI 478883]; *gum 2* = subsp. *gummifer* [Ames 26383]; *sat 1* = subsp. *sativus* (‘Amsterdam’); *sat 2* = subsp. *sativus* (‘Dolanka’); *sat 3* = subsp. *sativus* (DH1); *rou* = *D. rouyi*; *sah* = *D. sahariensis*; *syr* = *D. syrticus*; *orl* = *O. daucoides*

The UPGMA dendrogram based on six karyological parameters divided the 16 accessions into three major clusters at a Euclidean distance of 12.5, with a cophenetic correlation of 0.92 (Fig. 5). The first cluster, represented by *O. daucoides*, was separate, as a distinct outgroup, forming an independent branch. In the second cluster, *D. syrticus* and *D. aureus* were grouped together. The third cluster was subdivided into two subclusters, one of which comprised *D. sahariensis* and all *D. carota* subspecies, while the other one included only *D. rouyi*.

**Fig. 6:**
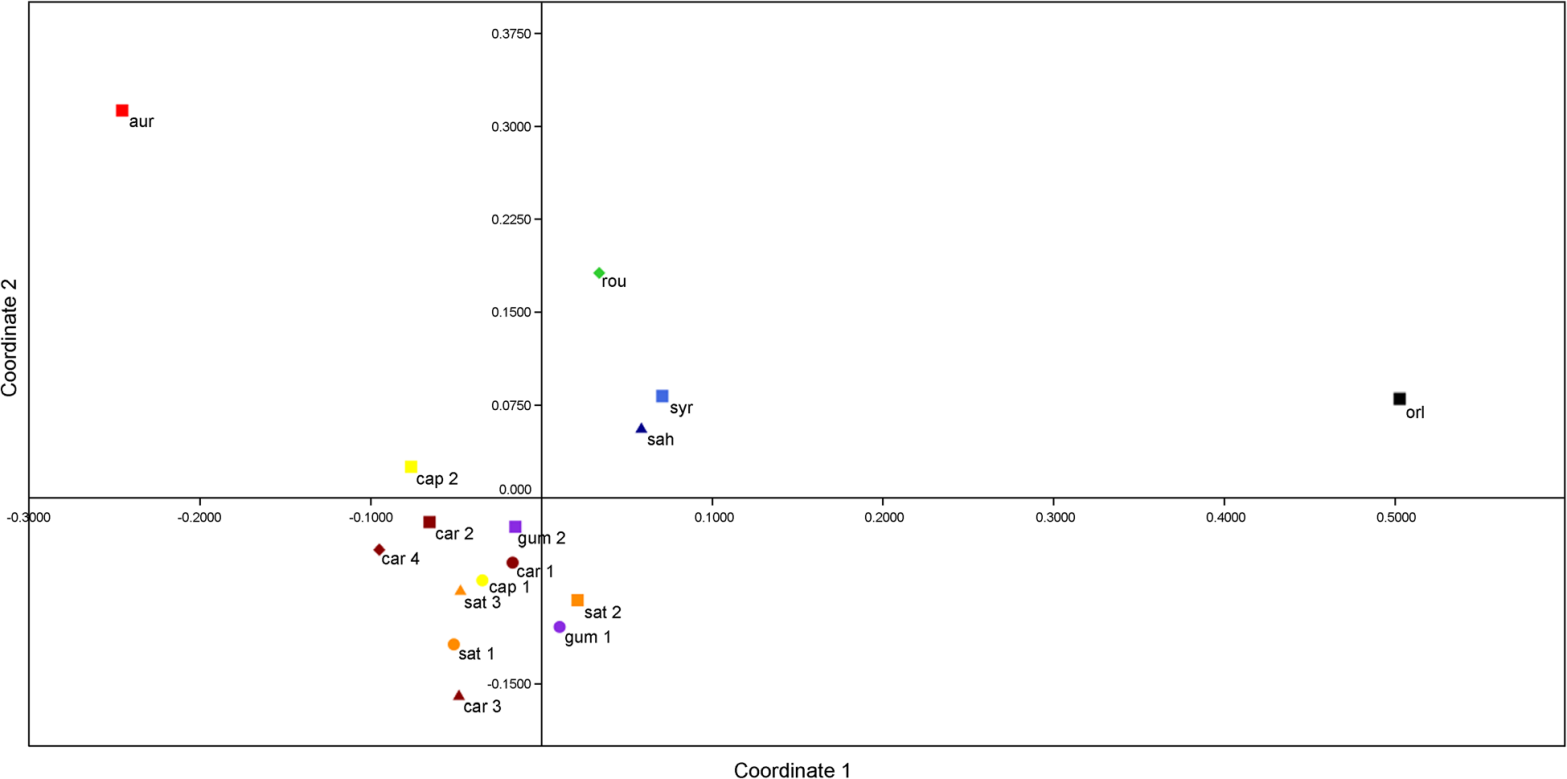
PCoA based on six quantitative parameters (*x*, 2*n*, THCL, M_CA_, CV_CL_, CV_CI_) for the 16 accessions of *Daucus* and *Orlaya daucoides*, which are listed in Table 2. *aur* = *D. aureus*; *cap 1* = *D. carota* subsp. *capillifolius* [PI 279764]; *cap 2* = subsp. *capillifolius* [Ames 30198]; *car 1* = subsp. *carota* [PI 274297]; *car 2* = subsp. *carota* [PI 478369]; *car 3* = subsp. *carota* [PI 478861]; *car 4* = subsp. *carota* [PI 652393]; *gum 1* = subsp. *gummifer* [PI 478883]; *gum 2* = subsp. *gummifer* [Ames 26383]; *sat 1* = subsp. *sativus* (‘Amsterdam’); *sat 2* = subsp. *sativus* (‘Dolanka’); *sat 3* = subsp. *sativus* (DH1); *rou* = *D. rouyi*; *sah* = *D. sahariensis*; *syr* = *D. syrticus*; *orl* = *O. daucoides*

Karyological relationships among the studied accessions revealed by PCoA are illustrated in Fig. 6. The results indicated that the first two principal coordinates explained 74% of the total variation. The PCoA scatter plot showed that all *D. carota* subspecies tended to cluster together, while three wild *Daucus* species, namely *D. sahariensis*, *D. syrticus*, and *D. rouyi*, were clearly separated from them. In contrast, *D. aureus* and *O. daucoides* occupied the most isolated positions, with *O. daucoides* being a distinct outgroup.

## Discussion

Karyotype features, especially chromosome number, chromosome length, karyotype asymmetry, the number of rDNA sites, and other chromosomal markers, are of great use in plant taxonomy and evolutionary studies. Thus, comparative karyotype analyses have been broadly utilized to elucidate relationships among taxa (at different taxonomic levels), as well as to understand the trends in chromosome evolution [41–47]. Moreover, comparative cytogenetic studies have provided evidence for extensive chromosome rearrangements in several plant species, e.g., those belonging to the families Brassicaceae [48, 49], Solanaceae [50, 51], and Poaceae [52–54]. The differences in karyotypes between related species, i.e., the chromosome number, shape, and structure, are caused by the syntenic groups that are assembled in different combinations. For example, groups that are fused together in one species may be separated on different chromosomes in another, or may be duplicated, inverted, or lost [55].

Centromeres are the key regions of eukaryotic chromosomes and are essential for sister chromatid cohesion. Additionally, centromeres are the sites where spindle microtubules attach via the kinetochore complex, thereby ensuring the proper segregation of chromosomes during cell division. Microscopically, they are recognizable on metaphase chromosomes as the primary constrictions and mostly contain large arrays of highly repetitive satellite DNA and retrotransposons [56, 57]. Typically, the monomers of centromeric satellite repeats range from 150 to 180 bp in length, e.g., pAL1 in *Arabidopsis* [58, 59], CentO in rice [60], CentC in maize [61], MtRs in *Medicago truncatula* [62], CL1 repeat in radish [16], CmSat162 in melon [14], and So1 in sugarcane [63]; however, longer monomers have also been reported [63–69]. Although the functional role of centromeres is conserved among all eukaryotes, the sequences of centromeric DNA and kinetochore proteins are considerably variable and evolve rapidly, even in closely related species, which is known as the ‘centromeric paradox’ [65, 70, 71]. For example, centromeres of rice (*Oryza sativa*) chromosomes contain a 155-bp satellite repeat CentO [60], whereas several wild *Oryza* species lack this sequence but instead contain different genome-specific centromeric satellite arrays [72, 73]. In potato (*Solanum tuberosum*), 12 centromeres show a large variation in terms of the structure and DNA composition, of which five centromeres lack satellite repeats but consist mainly of single- and low-copy sequences. In contrast, six potato centromeres contain megabase-sized arrays of satellite repeats, specific to individual centromeres; five of them appear to have emerged recently, since they were not found in the genomes of closely related *Solanum* species. In addition, most of these ‘young’ (newly emerged) centromeric repeats in potato were amplified from retrotransposon-related sequences [71]. Recently, Ávila Robledillo et al. [68] performed the largest study to date in terms of the number of related species investigated (14 species belonging to the legume tribe Fabeae) and newly centromeric satellites described. As a result, they found a great diversity of centromeric repeats within and between the analyzed Fabeae species. More recently, Huang et al. [63] discovered that some sugarcane centromeric satellites also exhibit high similarity with centromeric retrotransposons, indicating that they originated from these mobile elements. These repeats were flanked by direct repeats and formed extrachromosomal circular DNAs (eccDNAs). The retrotransposon-derived origin and the presence of eccDNAs elucidate how retrotransposons could evolve into centromeric satellites, providing new insights into the origin, formation pathways, and evolution of centromeric satellites in eukaryotes.

The original carrot centromeric repeat (CentDc) was isolated from BAC clone 004H08, which was initially selected for the *phytoene synthase 1* (*PSY1*) gene [39]. This BAC clone, as revealed by FISH, hybridized to the centromeric regions of all carrot chromosomes. Moreover, the FISH signals coincided with those produced by the carrot cot-1 DNA fraction, indicating that this BAC clone contained a dominant centromeric repeat [30]. As further evidenced by sequencing, the CentDc repeat unit of approximately 159 bp is composed of typically four 39–40-bp monomers (named A, B, C, and D) that vary slight in sequence, representing a higher-order repeat (HOR) structure [30, 33].

A comparative in silico analysis with some other *Daucus* species (from both *Daucus* I and II subclades) was also performed, revealing that the CentDc-like repeat represented the most abundant tandem repeat in the genomes of *D. syrticus* (named Ds-CL1) and *D. aureus* (named Da-CL1), varying, however, in terms of their structure. In *D. pusillus*, only the initial 40-bp monomer of its most abundant tandem repeat (named Dp-CL5) showed considerable similarity with monomer A of the original carrot CentDc element. These results indicate that these satellite families (CentDc, Ds-CL1, Da-CL1, and Dp-CL5) share a common evolutionary origin, predating the divergence of the two *Daucus* subclades. However, in two other species, *D. guttatus* and *D. littoralis*, CentDc-like sequences were not found, which was further confirmed by FISH [33].

To study the evolution of carrot centromeric satellite repeats, for comparative FISH analysis, we selected a number of *Daucus* taxa, including some cultivated carrots and several wild species and subspecies, as well as some closely related non-*Daucus* species. The studied accessions differed in terms of their chromosome number, geographical distribution, and phylogenetic position. As a result, we found that CentDc repeats were present in the genomes of several taxa of both *Daucus* subclades and one non-*Daucus* outgroup species (*Astrodaucus littoralis*), which indicates the ancient nature of CentDc, confirming the previous conclusion of Iorizzo et al. [33].

Moreover, our findings were also consistent with the results of the above-mentioned in silico analysis by Iorizzo et al. [33]. Out of the five species examined by the authors, we included four in our comparative FISH study, i.e., the same accessions of *D. aureus*, *D. littoralis*, *D. pusillus*, and *D. syrticus*, confirming the presence of CentDc-like sequences in the genomes of *D. aureus*, *D. pusillus*, and *D. syrticus* as well as the absence of these sequences in *D. littoralis*.

Although we found CentDc repeats in the genome of one outgroup species, this evidence is not enough to allow assumptions on the phylogeny of this species. With regard to the phylogenetic position of the outgroup species, according to Arbizu et al. [25], who examined 107 accessions of *Daucus* (92 accessions) and non-*Daucus* (15 accessions) taxa using DNA sequences of 94 nuclear orthologs, among the analyzed outgroup species, *Orlaya daucoides* and *O. daucorlaya* are sister to *Daucus*, whereas *Astrodaucus littoralis*, *Caucalis platycarpos*, *Turgenia latifolia*, *Torilis leptophylla*, *T. arvensis*, and *T. nodosa* are sister to all other examined taxa, and *Ammi visnaga* and *Oenanthe virgata* are sister to all of the above.

Although different *Daucus* taxa had various numbers and intensities of FISH signals, the CentDc repeats maintained the consistency of their centromeric localization. Such consistency in terms of chromosomal distribution of different satellites among closely related species has also been reported, e.g., in *Saccharum* (the centromeric satellite So1) [63] or radish and *Brassica* species (the subtelomeric satellite CL25) [16]. In the case of FISH-negative taxa, the absence of the CentDc repeats suggests that they may have been lost or replaced by different centromeric satellites or that their copy number was too low to be detected by means of molecular cytogenetics.

It should be noted, however, that, in this study, we used a 36-nucleotide FISH probe [31] based on the consensus sequence corresponding to a subrepeat of the original CentDc repeat [30, 39]. In the FISH-positive *Daucus* taxa, we determined the centromeric localization of these repeats using metaphase spreads with distinct primary constrictions (Additional file 4: Fig. S2), which is often challenging in *Daucus* and other species with small chromosomes, especially when the chromosomes are highly condensed. Although we also conducted FISH on both meiotic chromosomes and chromosomes in mitotic anaphase of selected accessions, it is essential to differentiate sequences truly associated with centromeric chromatin from other repetitive sequences. Thus, to ascertain the centromeric localization of CentDc repeats and comprehensively investigate the repeat composition of *Daucus* centromeres, it would be necessary to perform chromatin immunoprecipitation (ChIP) using antibodies against centromeric proteins (anti-CENH3), followed by sequencing of the immunoprecipitated DNA (ChIP-Seq). In addition, immunofluorescence experiments using an antibody against the carrot CENH3 protein (anti-DcCENH3 antibody) were performed in carrot and *D. glochidiatus* [74]. Combined localization of CENH3 and CentDc (immuno-FISH) has not yet been applied; however, such an approach should be considered in future research.

Of the FISH-positive taxa, the unique hybridization patterns of CentDc repeats in *D. aureus*, *D. conchitae*, *D. glochidiatus*, *D. involucratus*, and *D. muricatus* suggest that the CentDc probe may be used as a marker for the identification of these species. Moreover, in the case of *D. aureus*, *D. involucratus*, and *D. muricatus*, these findings were confirmed using different accessions of these species, which – except for *D. aureus* accessions – originated from different countries (see Table 1). However, to ascertain the species-specific FISH patterns of CentDc repeats for these species, it is necessary to analyze the remaining taxa from the genus *Daucus* that were not included in our study.

The haploid chromosome numbers of the majority of species within *Daucus* range between *n* = 8 and 11. One of the first reports on the somatic chromosome number of carrot (2*n* = 18) was published by Lindenbein [75], which was further confirmed by Sharma and Ghosh [35]. Only four other species, namely *D. annuus*, *D. insularis*, *D. sahariensis*, and *D. syrticus*, also have nine pairs of chromosomes [76, 77]. Most *Daucus* species are diploids with 2*n* = 20 or 22, yet some polyploids exist as well, i.e.*,* tetraploid *D. glochidiatus*, *D. incognitus*, *D. melananthos*, and *D. pedunculatus* (2*n* = 44) and hexaploid *D. montanus* (2*n* = 66) [78–80]. Here, we confirmed the previous chromosome counts for the investigated species; however, in the case of *D. conchitae*, to the best of our knowledge, we provided data on its somatic chromosome number for the first time.

Chromosome measurements may be precise only when the chromosomes are fully condensed and their boundaries are well defined. Thus, when analyzing chromosome spreads, it is crucial to exclude the ones containing either metaphase chromosomes that have not reached their maximum degree of condensation or prometaphase chromosomes. Moreover, chromosomes should have morphologically distinct primary constrictions; otherwise, it is difficult to determine the length of chromosome arms and, consequently, to calculate chromosomal parameters, such as centromeric index and karyotype asymmetry indices.

Karyotype asymmetry is an important karyotype character reflecting the general morphology of plant chromosomes and is thus widely used in comparative cytotaxonomy. A symmetrical karyotype comprises predominantly metacentric and submetacentric chromosomes of approximately equal size. Increased asymmetry may be caused either by the shifts in centromere position towards the telomeres (intrachromosomal) or by structural changes in chromatin (additions or deletions) that involve some chromosomes, leading to differences in the relative size between the chromosomes of the complement (interchromosomal) [41, 81, 82]. To date, several parameters and indices describing karyotype asymmetry have been proposed, including the quali-quantitative one proposed by Stebbins [40], as well as quantitative indices, of which Rec [83], A_2_ [84], R ratio [85], CV_CL_ [81] are measures of interchromosomal asymmetry, and TF% [86], AsK% [87], AsI% [88], Syi [83], A_1_ [84], A [89], CV_CI_ [81], and M_CA_ [90] characterize intrachromosomal asymmetry. Many of these parameters are, however, outdated and statistically incorrect, yet they are still widely used by a number of researchers [81, 90].

Since it is crucial to use only the parameters with a solid statistical basis for comparing karyotypes and reconstructing karyological relationships among taxa, here, we applied the methodology proposed by Peruzzi and Altınordu [91], considering six quantitative parameters (*x*, 2*n*, THCL, M_CA_, CV_CL_, CV_CI_) and subjecting them to principal coordinate analysis (PCoA), which is – thus far – the most legitimate approach to use. Our results showed that PCoA with these parameters was indeed a good way to establish the karyological relationships among taxa, as it clearly separated the wild *Daucus* species from the closely clustered *D. carota* subspecies. However, we observed some karyotypic variations between different accessions belonging to the same subspecies, which is especially noticeable in the UPGMA dendrogram, as they were placed into separate sub-subclusters. Moreover, the haploid karyotype formulae, to a large extent, also differed. This intrasubspecific diversity might be attributed to the different geographical distribution of these accessions, where different ecological, climatic, and altitude conditions occur. Similar observations were also made in some other taxa, e.g., *Dianthus* spp. [92] and *Zygophyllum fabago* [93], of which various geographically distant populations were sampled.

In terms of the Stebbins’ system, the karyotyped accessions were placed in 2A and 3A classes, indicating that these accessions have relatively symmetrical karyotypes (see Additional file 3: Table S2), and – from the evolutionary point of view – they are considered as primitive in this system [40].

Considering the karyotype of carrot, different researchers obtained different results in terms of karyotype formulae. Sharma and Ghosh [35], Sharma and Bhattacharyya [36], and Iovene et al. [29] observed a predominance of chromosomes with median and submedian primary constrictions for several cultivated forms of carrot. In contrast, our results resemble those described by Schrader et al. [38] and Nowicka et al. [32], who observed more asymmetrical karyotypes for different cultivated carrot forms. However, Schrader et al. [38] used Giemsa C-banded prometaphase chromosomes and did not specify the chromosome classification; on the other hand, works by Sharma and Ghosh [35] and Sharma and Bhattacharyya [36] had been published before Levan et al. [94] proposed a new (commonly used today) classification system; hence, these results are difficult to compare. Nevertheless, the observed discrepancies may be due to several reasons, including the line/cultivar/accession used, chromosome preparation methodology, or environmental conditions (e.g., climate, altitude). The latter may act as mutagenic factors, leading to changes in chromosome structure (deletions, additions), or may induce the activity of transposable elements; both of which cause variations in DNA content and, consequently, karyotype structure among accessions within a given species [95, 96]. However, the results on the effect of environmental factors on plant genomes have been inconclusive so far [95, 97, 98].

From the perspective of increasing human population, the need to increase carrot and other crops’ productivity is an actual challenge for researchers, inducing the development of breeding programs, that aim at obtaining new varieties that may be higher yielding, disease-resistant, or adapted to unfavorable conditions, especially in light of climate change and the alteration of natural ecosystems by human activities. The wild *Daucus* relatives may, therefore, play a significant role in the improvement of modern agriculture, providing genes that could be beneficial for breeding purposes, e.g., in adaptation to biotic and abiotic stresses, or climate change. In this context, a better understanding of the evolutionary relationships within the genus *Daucus* will contribute to future crop improvement programs [25, 79, 99].

## Conclusions

In this study, we determined the genomic organization of carrot centromeric repeats (CentDc) in 26 accessions of *Daucus* (belonging to both *Daucus* I and II subclades) and one accession of a closely related species. We showed that CentDc elements were present in the centromeric regions of all chromosomes of 20 accessions, representing 11 taxa, and thus can be used as centromere-specific cytogenetic markers. In the other *Daucus* taxa, the number of chromosome pairs with CentDc signals varied depending on the species, yet their centromeric localization was conserved. The presence of the CentDc repeats in the genomes of taxa belonging to both *Daucus* subclades and one outgroup species indicates the ancestral status of the repeat. In addition, we demonstrated the great usefulness of combining molecular cytogenetics with traditional chromosome measurements to study inter- and intraspecific karyological relationships among *Daucus* taxa.

Our observations provide useful information for further evolutionary, cytotaxonomic, and phylogenetic research on the genus *Daucus* and may contribute to a better understanding of the dynamic evolution of centromeric satellites in plants.

## Methods

### Plant material and chromosome preparation

In total, 34 accessions representing 22 taxa (species or subspecies), including 28 accessions from *Daucus* genus and 6 from closely related non-*Daucus* species, were selected for comparative FISH analysis (Table 1). Among the *Daucus* accessions, 12 were subspecies of *D. carota* (including one breeding line and two carrot cultivars) and 16 were from wild species belonging to *Daucus* subclades I and II. Seeds of all wild accessions were provided by the USDA-ARS North Central Regional Plant Introduction Station (Ames, Iowa, USA) and the Leibniz Institute of Plant Genetics and Crop Plant Research (IPK; Gatersleben, Germany), whereas the seeds of cultivated carrot were obtained either from the collections of the Department of Plant Biology and Biotechnology, University of Agriculture in Krakow (Krakow, Poland) or purchased from commercial sources.

The seeds were germinated either in soil-filled pots and grown under greenhouse conditions at 18 °C with a 16/8 h (light/dark) photoperiod or on moist filter paper in Petri dishes at 18 °C in the dark. Metaphase spreads were prepared from meristem root tip cells according to Nowicka et al. [31]. Root tips, approximately 1–2 cm in length, were collected from young plants or seedlings, pre-treated with 2 mM 8-hydroxyquinoline (Duchefa, Haarlem, the Netherlands) for 3.5 h at room temperature in the dark, and fixed in a freshly prepared mixture of methanol and glacial acetic acid (3:1, v/v) for at least 48 h. Meristems were then excised and digested in a mixture of 4% (w/v) cellulase Onozuka R-10. (Duchefa) and 2% (w/v) pectolyase Y-23 (Duchefa) in distilled water (pH 4.8) at 37 °C for 30–40 min. After digestion, the meristems were washed twice in distilled water, refixed in fixative, then macerated on a glass slide (one meristems per slide) using fine-pointed forceps, and flame-dried.

For meiotic preparations, flower buds at early stages of development were collected from greenhouse-grown plants of selected accessions and fixed in a freshly prepared mixture of ethanol and glacial acetic acid (3:1, v/v) for at least 48 h. After fixation, the flower buds were washed in a 10 mM citrate buffer (pH 4.8), and anthers were excised from the buds under a Leica S6D dissecting microscope (Leica Microsystems, Heerbrugg, Switzerland). The anthers were then digested in a mixture of 4% (w/v) cellulase Onozuka R-23, 2% (w/v) pectolyase Y-23, and 0.1% (w/v) cytohelicase (Sigma-Aldrich, St. Louis, USA) in 10 mM citrate buffer (pH 4.8) at 37 °C for 70–120 min. The digested anthers were washed twice in distilled water, refixed in fixative, then macerated on a glass slide (two anthers per slide) using fine-pointed forceps, and flame-dried.

To obtain cells in anaphase, a portion of the roots was not subjected to the treatment with 8-hydroxyquinoline but instead was fixed directly after collecting.

### DNA probe and fluorescence in situ hybridization

For comparative FISH mapping, we used a 36-nucleotide probe with the following sequence: 5′–ACTCGTTTGAAGTTGGAAACAACTTGTAGCTTCATT–3′ [31], which was designed and directly labeled with cyanine-5 (Cy5) at the 5′-end during synthesis by Genomed (Warsaw, Poland). The probe was based on the consensus sequence corresponding to a subrepeat of the previously described carrot centromeric repeat, named CentDc [30, 39]. Further, in this paper, we also refer to this 36-nucleotide sequence as CentDc.

The FISH procedure was carried out according to Czernicka et al. [100] with minor modifications. A hybridization mixture containing 50% (v/v) deionized formamide, 10% (w/v) dextran sulfate (Sigma-Aldrich), 2× SSC (0.3 M NaCl, 0.03 M Na_3_C_6_H_5_O_7_; pH 7.0), and 50 ng μL^− 1^ probe was denatured at 90 °C for 6 min, and instantly quenched in ice. The slides were denatured in 70% formamide/2× SSC at 80 °C for 1.5 min, immediately dehydrated in a graded ethanol series (70% ice-cold, 90 and 100% for 5 min each), and air-dried. The hybridization mixture was then applied to the slides, covered with a cover glass, sealed with rubber cement, and allowed to hybridize overnight at 37 °C in a humid chamber. After post-hybridization washes [2× SSC for 5 min, 2× SSC at 42 °C for 10 min, 2× SSC for 5 min, 1× PBS (0.13 M NaCl, 7 mM Na_2_HPO_4_, 3 mM NaH_2_PO_4_; pH 7.4) for 5 min], chromosomes were counterstained with 1 μg mL^− 1^ 4′,6-diamidino-2-phenylindole (DAPI) mounting medium (ProLong® Gold Antifade Mountant with DAPI; Thermo Fisher Scientific, Invitrogen™, Carlsbad, USA), and covered with a cover glass. For each accession, at least 3–5 plants were examined and thereby at least three independent FISH experiments per accession were performed.

The slides were examined under an Axio Imager.M2 fluorescence microscope (Carl Zeiss, Göttingen, Germany) equipped with the appropriate filter sets for DAPI (Zeiss filter set 02: λ_ex_ = 365 nm, λ_em_ > 420 nm) and Cy5 (Zeiss filter set 50: λ_ex_ = 640/30 nm, λ_em_ = 690/50 nm). The images were captured using a BV MV camera (Applied Spectral Imaging, Edingen-Neckarhausen, Germany) and Case Data Manager 6.0 software (Applied Spectral Imaging) and processed with FISHView® (Applied Spectral Imaging).

### Karyotype analysis

For karyotype analysis, 16 accessions, representing 9 taxa, were selected (for selection criteria, see 'Results'). For each accession, 4–10 well-spread mitotic metaphase plates were examined. Karyotypic parameters, including total haploid chromosome length (THCL) and chromosome length range (CLR), were determined using KaryoType 2.0 software [101]. Nomenclature used for the karyotype description followed that of Levan et al. [94]. To estimate karyotype asymmetry, the following karyotype asymmetry indices were used: CV_CL_ = coefficient of variation of chromosome length, CV_CI_ = coefficient of variation of centromeric index [81], and M_CA_ = mean centromeric asymmetry [90]; the formulae of these parameters are given in Additional file 2: Table S1. In addition, the accessions were categorized according to the karyotype symmetry classification of Stebbins [40] (Additional file 3: Table S2). For each karyotyped accession, a mean haploid idiogram was constructed by arranging the chromosomes in order of decreasing length.

To visualize karyotype asymmetry relationships among the studied accessions, a bidimensional scatter plot with parameters CV_CL_ vs. M_CA_ was drawn. To determine the karyological relationships among accessions, an unweighted pair-group method with arithmetic mean (UPGMA) cluster analysis with Euclidean distance and principal coordinate analysis (PCoA) using Gower’s similarity coefficient were performed based on six quantitative parameters (*x*, 2*n*, THCL, M_CA_, CV_CL_, CV_CI_), as proposed by Peruzzi and Altınordu [91]. Statistical analyses were performed using Past 3.22 software [102], and the UPGMA-based dendrogram and PCoA scatter plot were generated.

## Supplementary Information

**Additional file 1: Fig. S1.** FISH mapping of CentDc probe (red signals) to meiotic chromosomes (**a–g**) and chromosomes in mitotic anaphase (**h**) of selected *Daucus* accessions. **a** Pachytene chromosomes of *D. carota* subsp. *sativus* (‘Dolanka’); **b** DAPI-stained chromosomes from subpanel (**a**) that were digitally converted to a black-and-white image depicting cytologically recognizable heterochromatic knobs (*asterisks*), which CentDc signals coincide with, *arrowheads* indicate poorly visible knobs; **c** diakinesis chromosomes of Dolanka; **d** diakinesis and **e** metaphase I chromosomes of *D. aureus* [PI 319403], *arrows* indicate the chromosome pairs with additional CentDc signals; **f** pachytene chromosomes of *D. muricatus* [PI 295863], *arrow* indicates CentDc signals in the pericentromeric regions of one chromosome pair; **g** diakinesis chromosomes of *D. conchitae*, arrows indicate signals located at the most poleward positions of the chromosomes; **h** *D. pumilus*, CentDc signals located at the most poleward positions of the chromosomes in mitotic anaphase. Scale bar = 5 µm

**Additional file 2: Table S1.** Karyological parameters used in this study.

**Additional file 3: Table S2.** The classification of karyotypes in relation to their degree of asymmetry according to Stebbins (1971).

**Additional file 4: Fig. S2.** FISH mapping of CentDc probe (red signals) to the centromeric regions of metaphase chromosomes of selected *Daucus* accessions. **a** *D. carota* subsp. *carota* [PI 478369]; **b** subsp. *carota* [PI 274297]; **c** subsp. *sativus* (‘Dolanka’); **d** subsp. *capillifolius* [Ames 30198]; **e** subsp. *gummifer* [PI 478883]; **f** *D. aureus* [PI 319403]; **g** *D. muricatus* [PI 295863]; **h** *D. pumilus*; **i** *D. sahariensis* [Ames 29097]. Scale bar = 5 µm

## Data Availability

All data generated or analyzed during this study are included in this published article and its supplementary information files.
