# Supplementary figures and images for: Using carrot centromeric repeats to study karyotype relationships in the genus Daucus (Apiaceae)
Source: BMC Genomics. 2021 Jul 6;22:508. doi: 10.1186/s12864-021-07853-2 (PMC8259371; doi:10.1186/s12864-021-07853-2)

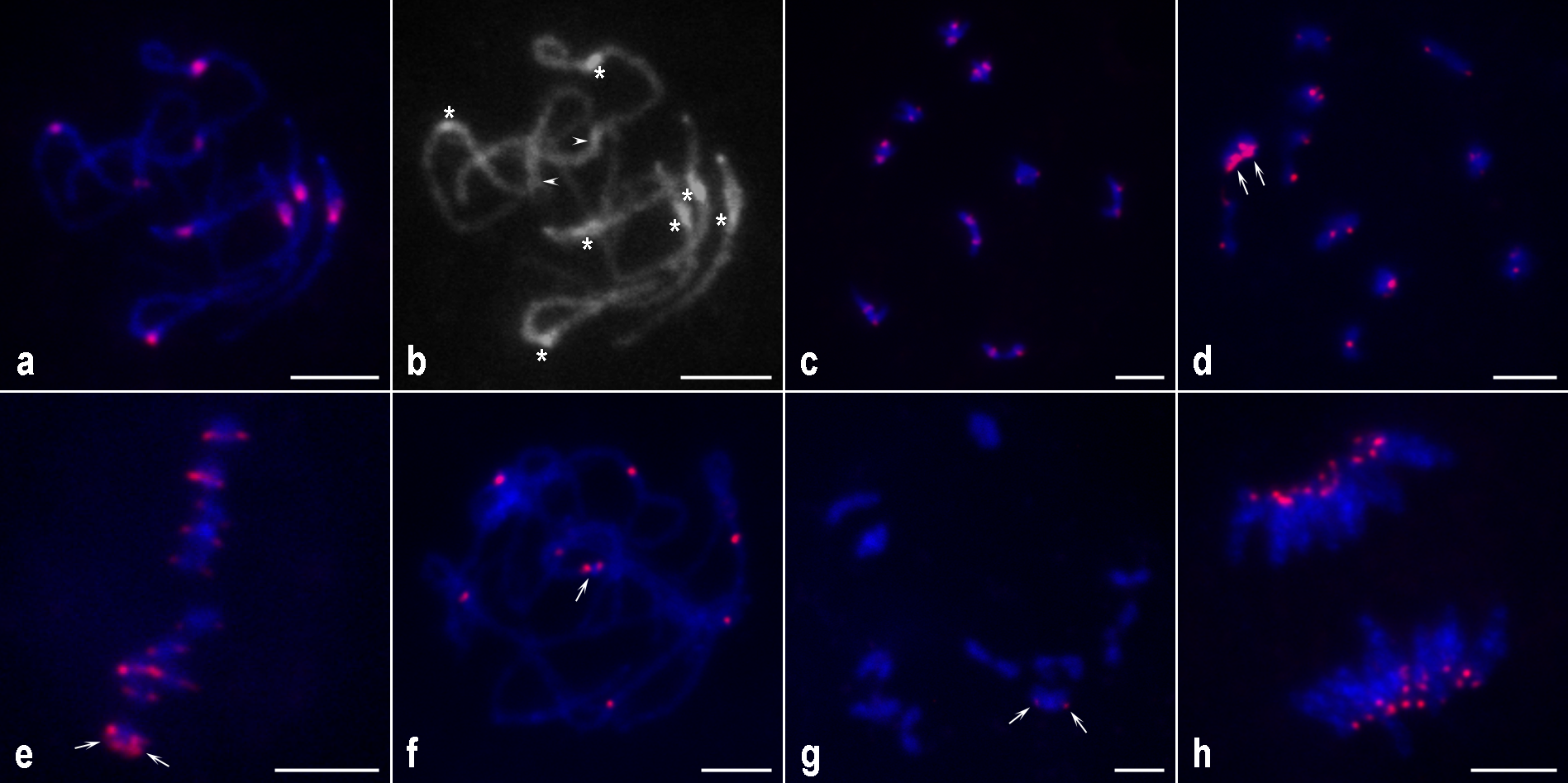

Supplement: Supplementary file 1 — Additional file 1: Fig. S1. FISH mapping of CentDc probe (red signals) to meiotic chromosomes (a–g) and chromosomes in mitotic anaphase (h) of selected Daucus accessions. a Pachytene chromosomes of D. carota subsp. sativus (‘Dolanka’); b DAPI-stained chromosomes from subpanel (a) that were digitally converted to a black-and-white image depicting cytologically recognizable heterochromatic knobs (asterisks), which CentDc signals coincide with, arrowheads indicate poorly visible knobs; c diakinesis chromosomes of Dolanka; d diakinesis and e metaphase I chromosomes of D. aureus [PI 319403], arrows indicate the chromosome pairs with additional CentDc signals; f pachytene chromosomes of D. muricatus [PI 295863], arrow indicates CentDc signals in the pericentromeric regions of one chromosome pair; g diakinesis chromosomes of D. conchitae, arrows indicate signals located at the most poleward positions of the chromosomes; h D. pumilus, CentDc signals located at the most poleward positions of the chromosomes in mitotic anaphase. Scale bar = 5 µm [file 12864_2021_7853_MOESM1_ESM.tif]

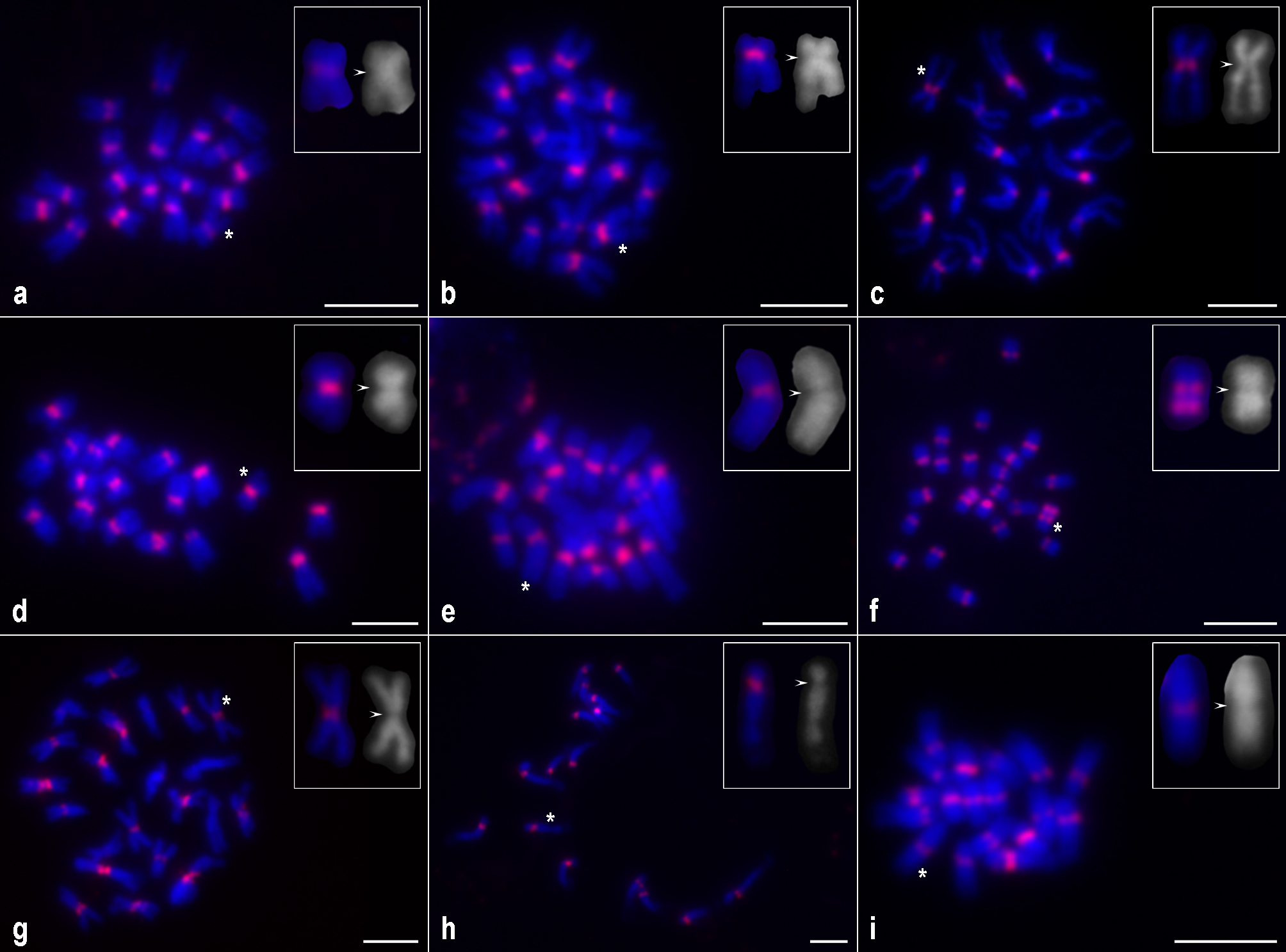

Supplement: Supplementary file 4 — Additional file 4: Fig. S2. FISH mapping of CentDc probe (red signals) to the centromeric regions of metaphase chromosomes of selected Daucus accessions. a D. carota subsp. carota [PI 478369]; b subsp. carota [PI 274297]; c subsp. sativus (‘Dolanka’); d subsp. capillifolius [Ames 30198]; e subsp. gummifer [PI 478883]; f D. aureus [PI 319403]; g D. muricatus [PI 295863]; h D. pumilus; i D. sahariensis [Ames 29097]. Scale bar = 5 µm [file 12864_2021_7853_MOESM4_ESM.tif]
