# Supplementary material for: Using carrot centromeric repeats to study karyotype relationships in the genus Daucus (Apiaceae)
Source: BMC Genomics. 2021 Jul 6;22:508. doi: 10.1186/s12864-021-07853-2 (PMC8259371; doi:10.1186/s12864-021-07853-2)
Supplement: Supplementary file 2 — Additional file 2: Table S1. Karyological parameters used in this study. [file 12864_2021_7853_MOESM2_ESM.docx]

**Table S1**. Karyological parameters used in this study

| Parameter | Abbreviation | Formula | Reference |
| --- | --- | --- | --- |
| Centromeric index  Chromosome length  Coefficient of variation of chromosome length  Degree of karyotype asymmetry  Mean centromeric asymmetry  Coefficient of variation of centromeric index | CI  CL  CV_CL_  A  M_CA_  CV_CI_ | S / (L + S)  L + S  (S_CL_ / X_CL_) × 100  Mean (L – S) / (L + S)  A × 100  (S_CI_ / X_CI_) × 100 | Paszko (2006)  Watanabe et al. (1999)  Peruzzi and Eroğlu (2013)  Paszko (2006) |

S = length of the short arm

L = length of the long arm

S_CL_ = the standard deviation of chromosome length

X_CL_ = mean chromosome length

S_CI_ = the standard deviation of the centromeric index

X_CI_ = mean centromeric index
