# Supplementary material for: Using carrot centromeric repeats to study karyotype relationships in the genus Daucus (Apiaceae)
Source: BMC Genomics. 2021 Jul 6;22:508. doi: 10.1186/s12864-021-07853-2 (PMC8259371; doi:10.1186/s12864-021-07853-2)
Supplement: Supplementary file 3 — Additional file 3: Table S2. The classification of karyotypes in relation to their degree of asymmetry according to Stebbins (1971). [file 12864_2021_7853_MOESM3_ESM.docx]

**Table S2**. The classification of karyotypes in relation to their degree of asymmetry according to Stebbins (1971)

| Chromosome ratio^a^ | Proportion of chromosomes with arm ratio < 2:1 | | | |
| --- | --- | --- | --- | --- |
|  | 1.00 (1) | 0.99–0.51 (2) | 0.50–0.01 (3) | 0.00 (4) |
| < 2:1 (A)  2:1–4:1 (B)  > 4:1 (C) | 1A  1B  1C | 2A  2B  2C | 3A  3B  3C | 4A  4B  4C |

^a^ The difference between the largest and the smallest chromosome of the complement
